# Supplementary material for: A random-walk benchmark for single-electron circuits
Source: Nat Commun. 2021 Jan 12;12:285. doi: 10.1038/s41467-020-20554-w (PMC7804119; doi:10.1038/s41467-020-20554-w)
Supplement: Supplementary file 1 — Supplementary Information [file 41467_2020_20554_MOESM1_ESM.pdf]

Supplemental Information for “A random-walk benchmark for single-electron circuits”

David Reifert,[1] Martins Kokainis,[2,3] Andris Ambainis,[2] Vyacheslavs Kashcheyevs,[3] and Niels Ubbelohde[1,*]

[1] *Physikalisch-Technische Bundesanstalt, 38116 Braunschweig, Germany*

[2] *Faculty of Computing, University of Latvia, 19 Raina boulevard, LV-1586 Riga, Latvia*

[3] *Department of Physics, University of Latvia, 3 Jelgavas street, LV-1004 Riga, Latvia*

CONTENTS

1. Baseline model 2
2. Estimation of step-wise probabilities $P^t_\pm$ by deconvolution 4
3. Comparison between measured and predicted $P_\pm$ 5
4. Model consistency and Fisher's significance testing 5
5. Consistency regions 6
6. Combining the p-values 7
7. Maximum likelihood estimation 7
8. Dirichlet distribution-based random-walk models 7
9. Fast-fluctuator model 8
10. Consistency of fast-fluctuator model 9
11. Slow-drift model 10
12. Consistency of slow-drift model 10
13. Excess noise simulations 11
14. Noise simulation results 13
15. Slow-drift model: variance of random walker's position 16
16. Proof for spread condition 19

SUPPLEMENTARY NOTE 1. BASELINE MODEL

Consider a time-homogeneous discrete-time random walk on the set of integers which starts at 0 and at each step moves $+1$ with probability P_+, moves -1 with probability P_- and stays at the same vertex with probability P_0; here we assume $P_+, P_-, P_0 \in (0,1)$, $P_- + P_0 + P_+ = 1$.

To describe this process formally, consider a random variable $\mathbf{K} = (K_{-1}, K_0, K_{+1})$ following a multinomial distribution with $t > 0$ trials and three categories, with associated probabilities P_-, P_0 and P_+, respectively. Then the random variable $X = K_{+1} - K_{-1}$ corresponds to the position of the random walker after t steps, since all steps can be modeled with independent discrete random variables with three possible outcomes (-1, 0 and $+1$, respectively) and respective probabilities P_-, P_0 and P_+. First we show that the probability mass function of the discrete variable $X \in \{-t, -t+1, \ldots, t-1, t\}$ is given by (1) in the main text; i.e., let $p_x^t := \Pr(X = x)$, then

Claim 1. *The probability mass function (PMF) of the variable X is*

$$p_x^t = \begin{cases} (P_+)^x (P_0)^{t-x} \binom{t}{x} \, {}_2F_1\left(\frac{x-t}{2}, \frac{x-t+1}{2}; x+1; \frac{4P_+P_-}{P_0^2}\right), & x \geq 0, \\ (P_-)^{-x} (P_0)^{t+x} \binom{t}{-x} \, {}_2F_1\left(\frac{-x-t}{2}, \frac{-x-t+1}{2}; -x+1; \frac{4P_+P_-}{P_0^2}\right), & x < 0, \end{cases} \tag{1}$$

for $x \in \{-t, -t+1, \ldots, t-1, t\}$.

Proof. Suppose that $x \geq 0$; then the event $X = x$, i.e., the event of the random walker being at the position x after t steps, is equivalent to the event that the multinomially distributed variable $\mathbf{K} = (K_{-1}, K_0, K_{+1})$ satisfies $K_{+1} - K_{-1} = x$ (i.e., to the event that the random walker has moved K_{+1} steps to the right and $K_{-1} = K_{+1} - x$ steps to the left). Therefore $\Pr(X = x)$ can be obtained from the multinomial distribution's PMF as

$$\Pr(X = x) = \sum_{\substack{\mathbf{k}: \\ k_1 - k_{-1} = x \\ k_{-1} + k_0 + k_1 = t}} \Pr(\mathbf{K} = \mathbf{k}).$$

Since $\mathbf{K}$ follows a multinomial distribution with $t > 0$ trials and three categories with respective probabilities P_-, P_0 and P_+, its PMF is given by

$$\Pr(\mathbf{K} = \mathbf{k}) = \frac{t!}{k_{-1}! k_0! k_1!} P_-^{k_{-1}} P_0^{k_0} P_+^{k_1},$$

where $\mathbf{k} := (k_{-1}, k_0, k_1)$ is a vector of nonnegative integers satisfying $k_{-1} + k_0 + k_1 = t$.

Notice that such $\mathbf{k}$ can additionally satisfy $k_1 - k_{-1} = x \geq 0$ only if $\mathbf{k}$ is of the form $\mathbf{k} = (s, t-x-2s, x+s)$, for some nonnegative integer s. Moreover, since $k_0 \geq 0$, we obtain the constraint $t - x - 2s \geq 0$, i.e., $s \leq 0.5(t-x)$. We conclude that all suitable vectors $\mathbf{k}$ are parametrized by a nonnegative integer s, which is upper-bounded by $0.5(t-x)$ (more precisely, the maximal valid s value is the floor function of $0.5(t-x)$). For each such s the corresponding vector is $\mathbf{k} = (s, t-x-2s, x+s)$ and the probability of the event $\mathbf{K} = \mathbf{k}$ is

$$\Pr(\mathbf{K} = (s, t-x-2s, x+s)) = \frac{t!}{s!(x+s)!(t-x-2s)!} P_-^s P_0^{t-x-2s} P_+^{x+s},$$

respectively. Thus $p_x^t := \Pr(X = x)$ is simply the sum of these multinomial probabilities:

$$\begin{aligned} p_x^t &= \sum_{\substack{\mathbf{k}: \\ k_1 - k_{-1} = x \\ k_{-1} + k_0 + k_1 = t}} \Pr(\mathbf{K} = \mathbf{k}) = \sum_{s=0}^{(t-x)/2} \Pr\left(\mathbf{K} = (s, t-x-2s, x+s)\right) \\ &= \sum_{s=0}^{(t-x)/2} \frac{t!}{s!(x+s)!(t-x-2s)!} P_-^s P_0^{t-x-2s} P_+^{x+s}. \end{aligned}$$

The latter quantity can be equivalently expressed as

$$p_x^t = P_+^x P_0^{t-x} \binom{t}{x} \sum_{s=0}^{(t-x)/2} \frac{(t-x)!}{(x+s)!(t-x-2s)!s!} \left(\frac{P_+P_-}{P_0^2}\right)^s.$$

Let us show that

$$\sum_{s=0}^{(t-x)/2} \frac{(t-x)!}{(x+s)!(t-x-2s)!s!} \left(\frac{P_+P_-}{P_0^2}\right)^s = {}_2F_1\left(\frac{x-t}{2}, \frac{x-t+1}{2}; x+1; \frac{4P_+P_-}{P_0^2}\right), \tag{2}$$

which will establish (1) and conclude the proof.

We start by rewriting the LHS of (2). Observe that $(x+s)! = (x+1)_s$, where $(a)_s := a(a+1)\dots(a+s-1)$ stands for the Pochhammer's symbol. Furthermore,

$$\begin{aligned}\frac{(t-x)!}{(t-x-2s)!} &= (t-x)(t-x-1)\dots(t-x+1-2s)\\ &= 4^s\left(\frac{t-x}{2}-s+1\right)_s\left(\frac{t-x-1}{2}-s+1\right)_s\\ &= 4^s\left(\frac{x-t}{2}\right)_s\left(\frac{x-t+1}{2}\right)_s,\end{aligned}$$

where the last step applies the identity $(-a)_s = (-1)^s(a-s+1)_s$. Therefore

$$\sum_{s=0}^{(t-x)/2} \frac{(t-x)!}{(x+s)!(t-x-2s)!s!} \left(\frac{P_+P_-}{P_0^2}\right)^s = \sum_{s=0}^{(t-x)/2} \frac{\left(\frac{x-t}{2}\right)_s\left(\frac{x-t+1}{2}\right)_s}{(x+1)_s\, s!} \left(\frac{4P_+P_-}{P_0^2}\right)^s.$$

The upper limit $(t-x)/2$ in the latter sum can be replaced with infinity, since the numerator $\left(\frac{x-t}{2}\right)_s\left(\frac{x-t+1}{2}\right)_s$ is zero for the additional terms with $s > 0.5(t-x)$. It remains to recognize now that the sum coincides with the definition of the Gaussian hypergeometric function ${}_2F_1$. We have verified (2), which concludes the proof of (1) when $x \geq 0$. The case $x < 0$ follows from similar considerations. □

We note that discrete distributions similar to X have been considered before. In particular, [1] considers an analogue of our random variable X and computes p_0^t (termed "return probability $p_0(t)$" in the paper). X is also closely related to the *inverse trinomial distribution* [2, 3], defined via a random walk on the line. Nevertheless, we are not aware of prior work establishing the PMF (1) of X.

The return probability for the random walk, p_0^t, is the probability of error-free electron transfer in the context of our benchmarking application, and hence can also be interpreted as transfer fidelity. As long as the contribution of the return paths is negligible, p_0^t decays exponentially, but for larger t the exponential decay is modified. Below we derive an explicit asymptotics that characterizes both sides of this crossover.

Claim 2.

$$p_0^t \approx \begin{cases} P_0^t, & t \ll (P_+P_-)^{-1/2} \\ \frac{\left(P_0+2\sqrt{P_+P_-}\right)^{1/2+t}}{(4\pi\, t)^{1/2}(P_+\, P_-)^{1/4}}, & t \gg (P_+P_-)^{-1/2} \end{cases}.$$

Proof. By (1) we have

$$p_0^t = (P_0)^t\, {}_2F_1\left(\frac{-t}{2}, \frac{-t+1}{2}; 1; z\right),$$

where we denote $z = 4P_+P_-/P_0^2$. We start by observing that by a quadratic transformation [4, Eq. 15.8.13] we have

$${}_2F_1\left(\frac{-t}{2}, \frac{-t+1}{2}; 1; z\right) = {}_2F_1\left(-t, 0.5; 1; \zeta\right)\cdot(1-0.5\zeta)^{-t}, \tag{3}$$

where the variable ζ is defined by $\frac{\zeta}{2-\zeta} = \sqrt{z}$, i.e.,

$$\zeta = \frac{2\sqrt{z}}{1+\sqrt{z}} = \frac{4\sqrt{P_+P_-}}{P_0+2\sqrt{P_+P_-}} \quad \text{and} \quad (1-0.5\zeta)^{-1} = \frac{P_0+2\sqrt{P_+P_-}}{P_0}.$$

Using the equality (3) we arrive at

$$p_0^t = \left(P_0+2\sqrt{P_+P_-}\right)^t\, {}_2F_1\left(-t, 0.5; 1; \zeta\right). \tag{4}$$

Since the hypergeometric function on the right hand side of (4) is a degree-t polynomial in the variable $\zeta \ll 1$, for small values of t we can approximate

$$P_0 + 2\sqrt{P_+ P_-} \approx P_0 \quad \text{and} \quad {}_2F_1\left(-t, 0.5; 1; \zeta\right) \approx 1,$$

leading to the first part of the claim.

Now we consider (4) with fixed ζ when $t \to +\infty$. We apply an asymptotic expansion of the hypergeometric function in case of a large argument due to Erdélyi [5, p. 77, Eq. 15], which exploits the relation between the (Gaussian) hypergeometric function ${}_2F_1$ and the confluent hypergeometric function ${}_1F_1$:

$${}_2F_1\left(-t, 0.5; 1; \zeta\right) \sim {}_1F_1(0.5; 1; -t\zeta) \sim \frac{\Gamma(1)(t\zeta)^{-0.5}}{\Gamma(0.5)}\left(1 + O\left(|t\zeta|^{-1}\right)\right) \sim \frac{1}{\sqrt{\pi t\zeta}}.$$

Combining this with (4) and substituting $\zeta = \frac{4\sqrt{P_+P_-}}{P_0+2\sqrt{P_+P_-}}$ gives us the second part of the claim. □

Finally, consider N independent observations of the random variable X, i.e., i.i.d. random variables $X_1, \ldots, X_N \sim X$. Let $Z_x = |\{j \in \{1, \ldots, N\} \;:\; X_j = x\}|$ be the number of times the value $x \in \{-t, \ldots, t\}$ appears among these N observations. Then the random variable

$$\mathbf{Z}_{N,t} = (Z_{-t}, Z_{-t+1}, \ldots, Z_0, Z_1, \ldots, Z_t)$$

follows a multinomial distribution with N trials and $2\,t+1$ categories, labeled from $-t$ to t, and respective probabilities p_x^t. When there is no ambiguity, this notation is simplified to $\mathbf{Z}$. The probability to observe a particular vector $\mathbf{z} \in \mathbb{N}_0^{2t+1}$, $\sum_{x=-t}^{t} z_x = N$ (where $\mathbb{N}_0$ stands for the set of nonnegative integers) is

$$\Pr(\mathbf{Z} = \mathbf{z}) = N! \prod_{x=-n}^{n} \frac{(p_x^t)^{z_x}}{z_x!}. \tag{5}$$

SUPPLEMENTARY NOTE 2. ESTIMATION OF STEP-WISE PROBABILITIES $P_\pm^t$ BY DECONVOLUTION

Under the assumption that the $P_\pm^t$-values are independent of the position x of the random walker, they can be extracted by deconvolution of p_x^t and p_x^{t+1}. For that let us expand the model used so far and consider a random walk on the set of integers which at time t performs transition $x \mapsto x + j$ with probability P_j^t, $x, j \in \mathbb{Z}$. Here $P_j^t \in (0, 1)$ for all j and $\sum_{j\in\mathbb{Z}} P_j^t = 1$.

The experiment yields two vectors from $\mathbb{R}^{2m+1}$, $m \in \mathbb{N}$, representing the distributions $\mathbf{p}^t = \left(p_{-m}^t, \ldots, p_{-1}^t, p_0^t, p_1^t, \ldots, p_m^t\right)$ and $\mathbf{p}^{t+1} = \left(p_{-m}^{t+1}, \ldots, p_{-1}^{t+1}, p_0^{t+1}, p_1^{t+1}, \ldots, p_m^{t+1}\right)$. We shall assume $P_j^t = 0$ for all $j \in \mathbb{Z}$ s.t. $|j| \geq m$. The distribution $\mathbf{p}^{t+1}$ represents the position of the random walker after $t + 1$ steps and satisfies

$$p_x^{t+1} = \sum_{j\in\mathbb{Z}} P_j^t p_{x-j}^t,$$

i.e., $\mathbf{p}^{t+1} = \mathbf{P}^t * \mathbf{p}^t$ is the discrete convolution of $\mathbf{P}^t = \left(P_{-m}^t, \ldots, P_{-1}^t, P_0^t, P_1^t, \ldots, P_m^t\right)$ and $\mathbf{p}^t$. Therefore $\mathbf{P}^t$ can be extracted by discrete deconvolution, which is performed as follows.

Let $\mathfrak{p}^{t+1}$, $\mathfrak{p}^t$ and $\mathfrak{P}^t$ stand for the Fourier transform of $\mathbf{p}^{t+1}$, $\mathbf{p}^t$ and $\mathbf{P}^t$, respectively, then

$$\mathfrak{p}^{t+1} = \mathfrak{P}^t \cdot \mathfrak{p}^t, \quad \text{i.e.,} \quad \mathfrak{P}_x^t = \frac{\mathfrak{p}_x^{t+1}}{\mathfrak{p}_x^t} \quad \text{for all } x.$$

The vector $\mathfrak{p}^t$ is calculated from p^t as

$$\mathfrak{p}_x^t = \sum_{n=-m}^{m} p_n^t \exp\left(-\mathrm{i}x\beta_n\right), \quad \text{where } \mathrm{i} = \sqrt{-1} \text{ and } \beta_n = \frac{2\pi n}{2m+1},$$

similarly for $\mathfrak{p}^{t+1}$. Now we can the get $\mathbf{P}^t$ by applying the inverse discrete Fourier transform to $\mathfrak{P}^t$:

$$P_j^t = \frac{1}{2m+1} \sum_{n=-m}^{m} \mathfrak{P}_n^t \exp\left(\mathrm{i}n\beta_j\right).$$

	single pumps			whole device	
m	$q_m^{(1)}$	$q_m^{(2)}$	x	P_x (measured)	P_x (predicted)
0	0.000 21(5)	$3.6(28) \times 10^{-5}$	−1	0.000 12(4)	0.000 21(5)
1	0.999 82(6)	0.999 975(19)	0	0.999 78(5)	0.999 80(6)
2	$0.0(20) \times 10^{-5}$	$0.0(12) \times 10^{-5}$	1	$0.0(21) \times 10^{-5}$	$3.6(34) \times 10^{-5}$

TABLE S1. Comparison between measured and predicted values of P_x, showing good agreement.

	single pumps			whole device	
m	$q_m^{(1)}$	$q_m^{(2)}$	x	P_x (measured)	P_x (predicted)
0	0.000 12(16)	0.000 00(14)	-1	0.239(6)	0.000 12(21)
1	0.999 74(19)	1.000 00(14)	0	0.776(5)	0.999 74(24)
2	0.000 12(17)	0.000 00(14)	1	0.000 12(16)	0.000 12(22)

TABLE S2. Disagreement between measured and predicted P_x-values for sharp-transient waveform.

Further details on how the deconvolution is performed and the uncertainty propagates can be found in [6]. Now that we have extracted $\mathbf{P}^t$, we find for the experiment described in the main text, that $P^t_{|j|>1} \approx 0$ which allows us to approximate $P^t_+ = P^t_{+1}$, and $P^t_- = P^t_{-1}$.

SUPPLEMENTARY NOTE 3. COMPARISON BETWEEN MEASURED AND PREDICTED $P_\pm$

The characterization of the single electron pumps gives us their transport statistic $q_m^{(i)}$, which is the probability of pump $i \in \{1, 2\}$ transporting $m \in \mathbb{Z}$ electrons. Assuming independence of simultaneous pump operation we can calculate the probability P_x that charge on the island increases by $x \in \mathbb{Z}$ electrons (here $P_{\pm 1}$ is equivalent to $P_\pm$ in (1) in the main text) as

$$P_x(\text{predicted}) = \sum_m q^{(1)}_{m+x} \cdot q^{(2)}_m \,. \tag{6}$$

Table S1 provides an example for agreement between measured and predicted values of $P_\pm$ for non-interacting pumps.

For the measurement in Table S2 the waveform of the pump drive was changed from a low-frequency sinusoidal to a sharp voltage transient. Here we see a strong disagreement between the prediction of single pump characterization and the measurement of the $P_\pm$-values. This disagreement is caused by a strong shift of the operation point which occurs as soon as the pumps are operated simultaneously, indicating a strong correlation between the pumps.

SUPPLEMENTARY NOTE 4. MODEL CONSISTENCY AND FISHER'S SIGNIFICANCE TESTING

The experimental data consist of observations (actually, rebinned observations as described in Supplementary Note 5) of random variables $\mathbf{Z}_{N_1,t_1}$, $\mathbf{Z}_{N_2,t_2}$, ..., $\mathbf{Z}_{N_L,t_L}$, for several different pairs (N_1, t_1), ..., (N_L, t_L), which, according to the model outlined in Supplementary Note 1, all share the same step probabilities (P_-, P_+).

We consider the problem of determining if there is a parameter $P_\pm$ such that the experimental data do not contradict the model, at the fixed significance level. More generally, we are interested in extracting a region in the parameter space such that the experimental data do not contradict the model for each choice of the parameter from the region; for brevity, we will refer to this region as *consistency region*. It should be stressed that this approach is different from *parameter estimation problem*, in that here we are interested in parameter values which cannot be statistically rejected as incompatible with the data, whereas the parameter estimation techniques deal with estimating the values of the parameters in some fashion, e.g., by finding the values of parameters under which the experimental data are most probable under the assumed model.

The problem of testing consistency of the model with a specific parameter value is twofold: since the data correspond to several pairs of (N, t), with different parameters N, t but the same step probabilities (P_-, P_+), there are two questions to be asked:

1. Are the data for the particular value of (N, t) consistent with the model for some parameter $P_\pm$?

2. Are all the data consistent with the model for some fixed value of $P_\pm$?

Let $\mathbf{z}_0$ be an observation of the random variable $\mathbf{Z} := \mathbf{Z}_{N,t}$, with prescribed parameters t, N but unknown probabilities P_-, P_0, P_+.

We employ Fisher's significance testing framework in order to extract the consistency regions for the parameter $\boldsymbol{\theta} = (P_-, P_0, P_+)$. In its simplest form, a Fisherian test formulates [7] a single hypothesis, the null hypothesis H_0, which specifies the null distribution (i.e., in our case $H_0 : \boldsymbol{\theta} = \boldsymbol{\theta}^*$ for some fixed $\boldsymbol{\theta}^*$); then a certain test statistic T is computed from the observation $\mathbf{z}_0$, leading to a value $T(\mathbf{z}_0)$. The p-value of the test is the tail probability of $T(\mathbf{Z})$ under H_0. In our setting, the test statistic will be non-negative and smaller values will indicate stronger disagreement with the null hypothesis. Then the p-value of the test is

$$p(\mathbf{z}_0) = \sum_{\substack{\mathbf{z}: \\ T(\mathbf{z}) \leq T(\mathbf{z}_0)}} \Pr(\mathbf{Z} = \mathbf{z}),$$

where $\Pr(\mathbf{Z} = \mathbf{z})$ stands for the probability of the event $\mathbf{Z} = \mathbf{z}$ under the null hypothesis and the sum is over all those values $\mathbf{z}$ of the random vector $\mathbf{Z}$ that satisfy $T(\mathbf{z}) \leq T(\mathbf{z}_0)$ In the Fisher's significance testing framework the p-value is interpreted as "a measure of extent to which the data do not contradict the model" [7, p.122]. Therefore Fisher's significance testing allows to check if H_0 must be rejected (at the chosen significance level) for the particular value $\boldsymbol{\theta}^*$; next, we shall employ Fisher's significance testing to extract the region of those $\boldsymbol{\theta}$ values for which the respective H_0 cannot be rejected, see Supplementary Note 5.

The problem of testing whether the parameters of a multinomial distribution equal specified values has been well-investigated [8–11]. The common approaches (such as Pearson's χ^2 test, G^2 test or power-divergence test [10] which subsumes the former tests) are asymptotic tests which can be highly biased. This is due to the fact that under the null hypothesis the random variable X has vanishingly small tail probabilities (and the actual observed samples $\mathbf{z}$ have zero observed counts in the respective positions). This phenomenon makes the asymptotic tests ill-suited for the actual data.

An alternative to the aforementioned tests is the exact multinomial test [10], which enumerates all possible multinomial outcomes; its test statistic T is the probability of obtaining the particular outcome under the null hypothesis. Then the p-value of the test is

$$\sum_{\substack{\mathbf{z}: \\ \Pr(\mathbf{Z}=\mathbf{z}) \leq \Pr(\mathbf{Z}=\mathbf{z}_0)}} \Pr(\mathbf{Z} = \mathbf{z}).$$

However, the exhaustive enumeration quickly becomes computationally intractable as N grows. We instead apply a Monte Carlo test (proposed in [12], see also [11, 13, 14]), which can be seen as an extension of the exact multinomial test. In the Monte Carlo hypothesis testing procedure, a large number (say, N_{sim}) samples from the multinomial distribution under the null hypothesis are simulated; for each sample $\mathbf{z}$ the test statistic $\Pr(\mathbf{z})$ is calculated (i.e., the probability to draw $\mathbf{z}$ from the distribution $\mathbf{Z}$ under the null hypothesis). Let k be the number of samples for which the test statistic is at least as extreme as for the observed vector $\mathbf{z}_0$ (i.e., the number of samples $\mathbf{z}$ for which $\Pr(\mathbf{z}) \leq \Pr(\mathbf{z}_0)$). Then the p-value of the test is $(k + 1)/(N_{\text{sim}} + 1)$.

SUPPLEMENTARY NOTE 5. CONSISTENCY REGIONS

Since $P_0 = 1 - P_+ - P_-$, the Monte Carlo tests are applied to extract 95% consistency region for the pair (P_-, P_+). This region is defined as the set of all admissible (P_-, P_+) values for which the p-value obtained by testing the hypothesis $H_0 : \boldsymbol{\theta} = (P_-, (1 - P_- - P_+), P_+)$ is at least 0.05.

In practice, since the observed vector $\mathbf{z}_0$ has many zero entries (as N is too small to observe "$X = x$" when $|x|$ is large) and, since the experimental data is limited to small $|x|$, the data are rebinned. Depending on the dynamical range of the detector and available computational resources, we consider a random variable $\tilde{\mathbf{Z}} = (\tilde{Z}_{-3}, \tilde{Z}_{-2}, \tilde{Z}_{-1}, \tilde{Z}_0, \tilde{Z}_1, \tilde{Z}_2, \tilde{Z}_3)$ instead of the random variable $\mathbf{Z}$ where

$$\tilde{Z}_{-3} = \sum_{x \leq -3} Z_x, \quad \tilde{Z}_3 = \sum_{x \geq 3} Z_x, \text{ and } \tilde{Z}_x = Z_x, \ |x| \leq 2, \tag{7}$$

and perform the aforementioned tests against an observation $\tilde{\mathbf{z}}_0$ of $\tilde{\mathbf{Z}}$. Further on, this subtlety will be assumed implicitly, i.e., when talking of the random variable $\mathbf{Z}$ or its observation $\mathbf{z}_0$, the rebinned counterparts $\tilde{\mathbf{Z}}$ and $\tilde{\mathbf{z}}_0$ are to be understood.

SUPPLEMENTARY NOTE 6. COMBINING THE p-VALUES

The discussion above attempts to answer if the data are consistent with some $P_\pm$, for a particular value of (N, t); the challenge now is to combine the statistical tests done for all L pairs of (N, t). While for each fixed pair (N, t) the 95% consistency region can be constructed from the observation of the respective $\mathbf{Z}_{N,t}$, the goal is to obtain a global measure of discrepancy between the data and the hypothesis $H_0 : \boldsymbol{\theta} = (P_-, (1 - P_- - P_+), P_+)$, taking into account the observations for all pairs (N, t).

This task can be viewed as the problem of combining several independent p-values, which arises in meta-analysis [15]. When testing a true point null hypothesis and the test statistic is absolutely continuous, it can be shown that the p-values under the null hypothesis are uniformly distributed in $[0, 1]$. This allows to apply, e.g., Fisher's method of testing uniformity [16] (for an overview of other ways to combine p-values, see [17, Appendix A]). In our case both the random variables $\mathbf{Z}_{N,t}$ and the test statistic are discrete, thus under the null hypothesis all p-values obtained for each pair (N, t) only approximate the uniform distribution. Fisher's method is used to approximately determine the combined p-value, even though in case of sparse discrete distribution this approximation may [18] yield conservative results.

In practice, due to the computational cost involved with computing the combined p-value, this global consistency test is only performed for a single value of $\boldsymbol{\theta}$. The value $(P_+, P_-) = (2.13 \times 10^{-5}, 6.92 \times 10^{-5})$ we performed the combined test on is the one under which the observed data are most probable, i.e., the maximum likelihood estimate, see Supplementary Note 7. However, the combined p-value 2.23×10^{-6} means that H_0 needs to be rejected; also visually (see Figure 2c, triangles) it is clear that the distribution of p-values is far from uniform. Hence one concludes that this model with fixed $P_\pm$ for all pairs (N, t) is incompatible with the experimental data.

SUPPLEMENTARY NOTE 7. MAXIMUM LIKELIHOOD ESTIMATION

The preceding discussion tries to determine if the data contradict the model, within the given level of significance. However, if one only tries to find the most suitable choice of parameters $P_\pm$, a natural approach is to maximize the likelihood function, i.e., (in case of a single observation for a single pair (N, t)) maximize the expression in (5), with z_x being the actual observed values, with respect to the unknown parameters $P_\pm$. The task is equivalent to maximizing the logarithm of the likelihood,

$$\ell_{N,t}(\boldsymbol{\theta}) = \ln \Gamma(N + 1) + \sum_{x=-n}^{n} \left(z_x \ln \left(p_x^t(\boldsymbol{\theta}) \right) - \ln \Gamma(z_x + 1) \right),$$

where $p_x^t(\boldsymbol{\theta})$ stands for the RHS in (1) and $\ln \Gamma$ is the natural logarithm of the gamma function.

Since the observations across the L different pairs (N_i, t_i) are assumed to be independent, the joint probability of observing the complete data is the product of individual probabilities for each separate (N_i, t_i), i.e., the global log-likelihood function to be maximized is

$$\ell(\boldsymbol{\theta}) = \sum_{i=1}^{L} \ell_{N_i,t_i}(\boldsymbol{\theta}).$$

Maximizing this function over the standard 2-simplex using the experimental data gives the maximum likelihood estimate $(P_+, P_-) = (2.13 \times 10^{-5}, 6.92 \times 10^{-5})$.

SUPPLEMENTARY NOTE 8. DIRICHLET DISTRIBUTION-BASED RANDOM-WALK MODELS

Further we consider the case when the step probabilities P_-, P_+ are themselves random variables. We assume that (P_-, P_0, P_+) follows a Dirichlet distribution, which is [19] "one of the key multivariate distributions for random vectors confined to the simplex". The Dirichlet distribution also becomes important when the observed data are superficially

similar to the multinomial distribution but exhibit more variance than the multinomial distribution permits. As authors in [19, p.199] note, "One possibility of this kind of extra variation is that the multinomial probabilities" are not constant across the trials and the vector of probabilities can be interpreted as a random vector in the standard simplex; in this case the Dirichlet distribution is a convenient choice, resulting in a compound probability distribution, the Dirichlet-multinomial distribution [19, Definition 6.1].

The Dirichlet distribution on the standard 2-simplex Δ^2 with positive parameter vector $\boldsymbol{\alpha} = (\alpha_0, \alpha_1, \alpha_2)$, denoted by $\mathrm{Dir}(\boldsymbol{\alpha})$, is a probability distribution with [19, Definition 2.1] the density function

$$f_{\boldsymbol{\alpha}}(\boldsymbol{\theta}) = \frac{\prod_{i=0}^{2} \Gamma(\alpha_i)}{\Gamma(\sum_{i=0}^{2} \alpha_i)} \theta_0^{\alpha_0 - 1} \theta_1^{\alpha_1 - 1} \theta_2^{\alpha_2 - 1}, \quad \boldsymbol{\theta} = (\theta_0, \theta_1, \theta_2) \in \Delta^2. \tag{8}$$

The mean value and the variance of θ_i, $i = 0, 1, 2$, are given by

$$\mathrm{E}(\theta_i) = \frac{\alpha_i}{\sum_{j=0}^{2} \alpha_j} =: \tilde{\alpha}_i, \quad \mathrm{Var}(\theta_i) = \frac{\tilde{\alpha}_i (1 - \tilde{\alpha}_i)}{1 + \sum_{j=0}^{2} \alpha_j},$$

respectively, i.e., the mean value of θ_i is proportional to the parameter α_i, but the variance of θ_i decreases as $\sum_{i=0}^{2} \alpha_i$ is increased. This allows to employ the Dirichlet distribution to model the scattering of the vector $(P_-, P_0, P_+) \in \Delta^2$ around its mean value with a single additional parameter characterizing the magnitude of the scattering.

We proceed by considering two extensions of the baseline model, one where the variable $\boldsymbol{\theta} = (P_-, P_0, P_+)$ is chosen independently for each of the N separate random walks, and another where $\boldsymbol{\theta} = (P_-, P_0, P_+)$ is the same for all N random walks (but another $\boldsymbol{\theta} \sim \mathrm{Dir}(\boldsymbol{\alpha})$ is independently drawn if either N or t is changed).

SUPPLEMENTARY NOTE 9. FAST-FLUCTUATOR MODEL

Model description

Let $\boldsymbol{\alpha} = (\alpha_{-1}, \alpha_0, \alpha_1)$ be a fixed vector of positive parameters. For each pair (N, t) we consider the following process:

- repeat N times:
  - choose a random vector $\boldsymbol{\theta} = (P_-, P_0, P_+) \sim \mathrm{Dir}(\boldsymbol{\alpha})$ (independently each time);
  - perform t steps of the random walk with the respective step probabilities (P_-, P_0, P_+);
  - observe the position of the random walker $X \in \{-t, -t+1, \dots, t-1, t\}$;
- given the N observations $X_1, \dots, X_N$, denote $Z_x = |\{j \in \{1, \dots, N\} \, : \, X_j = x\}|$ and define the random variable

$$\mathbf{Z}_{N,t} = (Z_{-t}, Z_{-t+1}, \dots, Z_0, Z_1, \dots, Z_t).$$

This model corresponds to choosing step probabilities P_-, P_+ independently for each repetition of a random walk of a fixed length t. This way the random variable $\mathbf{Z}_{N,t}$ again has multinomial distribution, but now with modified (compared to the baseline model) probabilities incorporating the underlying Dirichlet distribution.

Probability mass function

To describe this process more formally, for each pair (N, t) let $\mathbf{K} = (K_{-1}, K_0, K_{+1})$ have the Dirichlet-multinomial distribution with $t > 0$ trials and parameter $\boldsymbol{\alpha} = (\alpha_{-1}, \alpha_0, \alpha_1)$. Define a random variable $X = K_{+1} - K_{-1}$, supported in the set $\{-t, -t+1, \dots, t-1, t\}$; denote $p_x^t := \Pr(X = x)$ and define a multinomial variable $\mathbf{Z}_{N,t}$ with N trials, $2t+1$ categories (from $-t$ to t) and the respective probabilities p_x^t, $x \in \{-t, -t+1, \dots, t-1, t\}$.

Since $\mathbf{K}$ follows the Dirichlet-multinomial distribution, its PMF (for a vector of nonnegative integers $\mathbf{k} = (k_{-1}, k_0, k_1)$ s.t. $k_{-1} + k_0 + k_1 = t$) satisfies [20, Eq. 35.152]

$$\Pr(\mathbf{K} = \mathbf{k}) = \frac{t! \, \Gamma(\alpha_\bullet)}{\Gamma(t + \alpha_\bullet)} \frac{\Gamma(k_{-1} + \alpha_{-1}) \Gamma(k_0 + \alpha_0) \Gamma(k_1 + \alpha_1)}{k_{-1}! \, k_0! \, k_1! \Gamma(\alpha_{-1}) \Gamma(\alpha_0) \Gamma(\alpha_1)},$$

where we denote $\alpha_\bullet := \sum_i \alpha_i$. Notice that if we keep the fractions $\theta_i := \frac{\alpha_i}{\alpha_\bullet}$ fixed, then in the limit $\alpha_\bullet \to \infty$ the random variable $\mathbf{K}$ becomes multinomially distributed, i.e.,

$$\Pr(\mathbf{K} = \mathbf{k}) \xrightarrow[\alpha_\bullet \to \infty]{} \frac{t!}{k_{-1}!\, k_0!\, k_1!} \theta_{-1}^{k_{-1}} \theta_0^{k_0} \theta_1^{k_1}.$$

This follows easily from the gamma function property $\Gamma(k+a) \sim \Gamma(a) a^k$ as $a \to \infty$.

Henceforth,

$$\begin{aligned} p_x^t &= \sum_{\substack{\mathbf{k}: \\ k_1 - k_{-1} = x \\ k_{-1} + k_0 + k_1 = t}} \Pr(\mathbf{K} = \mathbf{k}) = \sum_{l = \max\{0, -x\}}^{(t-x)/2} \Pr\left(\mathbf{K} = (l, t - x - 2l, x + l)\right) \\ &= \frac{t!\, \Gamma(\alpha_\bullet)}{\Gamma(t + \alpha_\bullet) \prod_i \Gamma(\alpha_i)} \sum_{l = \max\{0, -x\}}^{(t-x)/2} \frac{\Gamma(l + \alpha_{-1}) \Gamma(t - x - 2l + \alpha_0) \Gamma(x + l + \alpha_1)}{l! (t - x - 2l)! (x + l)!}. \end{aligned} \tag{9}$$

Observe that keeping the fractions $\frac{\alpha_i}{\alpha_\bullet}$ fixed and letting $\alpha_\bullet \to \infty$ makes the probabilities p_x^t given by (9) tend to the respective probabilities given by (1) (with $P_- = \alpha_{-1}/\alpha_\bullet$ and $P_+ = \alpha_1/\alpha_\bullet$).

After N independent observations the multinomial vector $\mathbf{Z}_{N,t}$ is obtained, supported in the set $\left\{ \mathbf{z} \in \mathbb{N}_0^{2t+1} : \sum_{x=-t}^{t} z_x = N \right\}$, with

$$\Pr(\mathbf{Z}_{N,t} = \mathbf{z}) = N! \prod_{x=-n}^{n} \frac{(p_x^t)^{z_x}}{z_x!}, \quad \mathbf{z} \in \mathbb{N}_0^{2t+1}, \ \sum_{x=-t}^{t} z_x = N. \tag{10}$$

The variable $\mathbf{Z}_{N,t}$ still has the multinomial distribution, as in the baseline model, and Eq. (10) is the same as (5) but with p_x^t given by (9). However, in contrast to the baseline model, the vector $\mathbf{K}$ has the Dirichlet-multinomial distribution instead of the multinomial distribution as before. That, in turn, implies that the probabilities p_x^t are not calculated from (1), but given by (9) instead. In effect, $\mathbf{Z}_{N,t}$ is a multinomial distribution, but different probabilities associated with its categories, when compared to the baseline model.

SUPPLEMENTARY NOTE 10. CONSISTENCY OF FAST-FLUCTUATOR MODEL

Consistency of this model is tested similarly as in the baseline case:

- For each particular pair (N, t), we perform a Fisherian test of the hypothesis $H_0 : \boldsymbol{\alpha} = \boldsymbol{\alpha}^*$ for some fixed $\boldsymbol{\alpha}^*$, given an observation $\mathbf{z}_0$ of $\mathbf{Z} = \mathbf{Z}_{N,t}$. The test is again conducted in the Monte Carlo manner as described previously, by drawing N_{sim} samples from the multinomial distribution under the null hypothesis and extracting the p-value as $(k+1)/(N_{\text{sim}}+1)$. Here k indicates the number of the simulated samples $\mathbf{z}$ satisfying $\Pr(\mathbf{Z} = \mathbf{z}) \leq \Pr(\mathbf{Z} = \mathbf{z}_0)$.
- Consistency of the model taking into account all L different pairs (N, t) is done by combining the L obtained p-values, via Fisher's method of testing uniformity.

The value $\boldsymbol{\alpha}^*$ to be tested in the previous step is again the maximum likelihood estimate, obtained by maximizing the function

$$\ell(\boldsymbol{\alpha}) = \sum_{i=1}^{L} \ell_{N_i, t_i}(\boldsymbol{\alpha}),$$

where

$$\ell_{N,t}(\boldsymbol{\alpha}) = \ln \Gamma(N+1) + \sum_{x=-n}^{n} \left(z_x \ln \left(p_x^t(\boldsymbol{\alpha}) \right) - \ln \Gamma(z_x + 1) \right),$$

and $p_x^t(\boldsymbol{\alpha})$ is given by the RHS of (9). Maximizing this function over the parameter space using the experimental data gives the maximum likelihood estimate $\boldsymbol{\alpha}^* = (9.08 \times 10^1, 1.31 \times 10^6, 2.78 \times 10^1)$. However, the combined p-value 2.08×10^{-6} again indicates that H_0 needs to be rejected; as it is seen in Figure 2c (squares), the distribution of p-values still remains far from uniform. Consequently, this model is also incompatible with the experimental data.

SUPPLEMENTARY NOTE 11. SLOW-DRIFT MODEL

Model description

Let again $\boldsymbol{\alpha} = (\alpha_{-1}, \alpha_0, \alpha_1)$ be a vector of positive parameters. Now we consider the following process for each pair (N, t):

- choose a random vector $\boldsymbol{\theta} = (P_-, P_0, P_+) \sim \mathrm{Dir}(\boldsymbol{\alpha})$;
- repeat N times:
  - perform t steps of the random walk with the respective step probabilities (P_-, P_0, P_+);
  - observe the position of the random walker $X \in \{-t, -t+1, \ldots, t-1, t\}$;
- given the N observations $X_1, \ldots, X_N$, denote $Z_x = |\{j \in \{1, \ldots, N\} \ : \ X_j = x\}|$ and define the random variable

$$\mathbf{Z}_{N,t} = (Z_{-t}, Z_{-t+1}, \ldots, Z_0, Z_1, \ldots, Z_t).$$

This way, the vector $\boldsymbol{\theta} = (P_-, P_0, P_+) \sim \mathrm{Dir}(\boldsymbol{\alpha})$ is drawn independently across different pairs (N, t), yet for each particular (N, t) it is fixed for all N random walks (the N random walks are assumed to be conditionally independent given $\boldsymbol{\theta}$). The resulting random variable $\mathbf{Z}_{N,t}$ has a discrete compound distribution, akin to the Dirichlet-multinomial distribution; however, $\mathbf{Z}_{N,t}$ is not multinomially distributed anymore.

Technically, the key difference from the previous model is that all N random walks use the same (randomly drawn from $\mathrm{Dir}(\boldsymbol{\alpha})$) vector $\boldsymbol{\theta}$, therefore marginalization of $\boldsymbol{\theta}$ happens only after forming the counts vector $\mathbf{Z}_{N,t}$. In contrast, in the previous model the Dirichlet variable is marginalized after forming the vector $\mathbf{K}$, resulting in the Dirichlet-multinomial distribution for $\mathbf{K}$ and a standard multinomial variable $\mathbf{Z}_{N,t}$.

Probability mass function

To characterize the model more formally, for each pair (N, t) and a fixed vector $\boldsymbol{\theta} = (P_-, P_0, P_+)$ let $p_x^t(\boldsymbol{\theta})$, $|x| \le t$, be defined as in the RHS of (1). The random variable $\mathbf{Z}_{N,t}$ is defined by compounding the multinomial distribution (5) with the Dirichlet distribution $\mathrm{Dir}(\boldsymbol{\alpha})$, i.e., $\mathbf{Z}_{N,t}$ is supported in the set $\left\{\mathbf{z} \in \mathbb{N}_0^{2t+1} : \sum_{x=-t}^{t} z_x = N\right\}$ and its PMF is obtained by marginalizing over the Dirichlet variable: for $\mathbf{z} \in \mathbb{N}_0^{2t+1}$ such that $\sum_{x=-t}^{t} z_x = N$,

$$\Pr(\mathbf{Z}_{N,t} = \mathbf{z}) = \frac{N!}{\prod_{x=-n}^{n} z_x!} \int_{\Delta^2} \prod_{x=-n}^{n} \left(p_x^t(\boldsymbol{\theta})\right)^{z_x} f_{\boldsymbol{\alpha}}(\boldsymbol{\theta})\, \mathrm{d}\boldsymbol{\theta}, \tag{11}$$

where the integration is over the standard 2-simplex Δ^2 and

$$f_{\boldsymbol{\alpha}}(\boldsymbol{\theta}) = \frac{\prod_{i=-1}^{1} \Gamma(\alpha_i) \theta_i^{\alpha_i - 1}}{\Gamma(\alpha_\bullet)}, \qquad \alpha_\bullet := \alpha_{-1} + \alpha_0 + \alpha_1, \tag{12}$$

is the PDF of the Dirichlet distribution (adapted from (8)). It is worth mentioning that since only the parameters $P_\pm, P_0$ are chosen from the Dirichlet distribution, instead of all $2t+1$ event probabilities associated to the multinomial distribution, the resulting compound distribution is not Dirichlet-multinomial.

SUPPLEMENTARY NOTE 12. CONSISTENCY OF SLOW-DRIFT MODEL

Given an observation $\mathbf{z}_0$ of $\mathbf{Z}_{N,t}$, we again perform Monte Carlo test of the hypothesis $H_0 : \boldsymbol{\alpha} = \boldsymbol{\alpha}^*$, for some fixed $\boldsymbol{\alpha}^*$. However, now the probability $\Pr(\mathbf{Z}_{N,t} = \mathbf{z})$ has the complicated analytical form (11), which is difficult to compute numerically. Therefore also $\Pr(\mathbf{Z}_{N,t} = \mathbf{z})$ is estimated via Monte Carlo approximation, i.e., for the particular parameter $\boldsymbol{\alpha}^*$ and the observed vector $\mathbf{z}_0$ we

- draw N_{sim} independent samples $\boldsymbol{\theta} \in \Delta^2$ from $\mathrm{Dir}(\boldsymbol{\alpha}^*)$;

- for each of the sampled vectors $\boldsymbol{\theta} = (P_-, P_0, P_+)$ draw a sample $\mathbf{z}$ from the multinomial distribution specified by (5) (where the probabilities p_x^t are computed using the sampled values P_-, P_+).

- This way N_{sim} vectors $\mathbf{z}_1, \ldots, \mathbf{z}_{N_{\text{sim}}}$ are obtained, among them many may coincide. Suppose that m distinct vectors $\mathbf{z}'_1, \ldots, \mathbf{z}'_m$ were obtained, with their respective frequencies $k_1, k_2, \ldots, k_m$, $\sum_i k_i = N_{\text{sim}}$. We can assume that $k_1 \leq k_2 \leq \ldots \leq k_m$.

- Suppose that $\mathbf{z}'_j$ coincides with the actual observation $\mathbf{z}_0$, and (provided that $j < m$) $k_j < k_{j+1}$; then the p-value of the test is declared $(k+1)/(N_{\text{sim}}+1)$, where $k := k_1 + k_2 + \ldots + k_j$. In case $\mathbf{z}_0$ does not occur among the N_{sim} obtained vectors, the p-value is declared 0.

By employing the outlined procedure, we can perform consistency testing similarly as before. Consistency of the model taking into account all L different pairs (N, t) is done by combining the L obtained p-values, via Fisher's method of testing uniformity.

The value $\boldsymbol{\alpha}^*$ to be tested now is found differently, compared to the previous models. This is due to the fact that the probabilities $\Pr(\mathbf{Z} = \mathbf{z})$ are estimated only approximately via Monte Carlo, which complicates maximizing the likelihood function.

Instead, we fix the fractions $\frac{\alpha_i}{\alpha_\bullet}$ to the best values of $P_\pm$ found in the baseline model (Supplementary Note 7) and optimize the parameter $\alpha_\bullet$, i.e., $\boldsymbol{\alpha}$ is in form $\alpha_\bullet \cdot\ (P_-, P_0, P_+)$, where $(P_+, P_-) = (2.13 \times 10^{-5}, 6.92 \times 10^{-5})$. The cost function associated with $\alpha_\bullet$ is

$$C(\alpha_\bullet) = \sum_{i=1}^{L} \left| p_{(i)} - \frac{i}{L} \right|, \tag{13}$$

where $p_{(i)}$ stands for the i-th smallest value among $p_1, p_2, \ldots, p_L$, where the latter are the p-values returned by the L tests of the hypothesis $H_0 : \boldsymbol{\alpha} = \alpha_\bullet \cdot\ (P_-, P_0, P_+)$. In other words, the cost function measures the distance between the empirical distribution function of p-values and the line corresponding to the cumulative distribution function corresponding to the uniform distribution.

The minimization of the cost function over $\alpha_\bullet$ estimates the optimal parameter to $\boldsymbol{\alpha}^* = (2.43 \times 10^3, 3.50 \times 10^7, 7.47 \times 10^2)$, with precision limited by numerical expense of Monte Carlo trials for $p_{(i)}$. The combined p-value equals 0.71, therefore the null hypothesis $H_0 : \boldsymbol{\alpha} = \boldsymbol{\alpha}^*$ cannot be rejected. Also, as it is seen in Figure 2c (diamonds), the distribution of p-values visually conforms to the uniform. Henceforth, the experimental data do not contradict this model.

SUPPLEMENTARY NOTE 13. EXCESS NOISE SIMULATIONS

The purpose of this Supplementary Note is to illustrate that our excess noise models can be consistent with and give reasonable estimates of realistic parametric variability one expects from an ensemble of TLFs (multi-timescale $1/f$-noise), or from a single but strong TLF (bimodal excess noise), despite a generic Dirichlet distribution and a single correlation timescale underpinning the "fast-fluctuator" and the "slow-drift" tests.

First we define a model that simulates fluctuating environment of the real experiments, and then analyze the simulated error counts using the same statistical tests as applied in the main text and described in Supplementary Notes 4, 9 and 11. The results in Section 14 below illustrate the three main findings summarized in the main text: (i) a threshold in excess noise amplitude, above which the advanced statistical tests become useful; (ii) an example of simulated environment with results of statistical tests similar to experiment, and a comparison between estimated and actual measure of parametric variability $\Delta P_\pm$; (iii) a summary of single fluctuator behavior w.r.t. "fast fluctuator" and "slow drift" statistical test.

Our approach to simulate $1/f$ noise by an ensemble of two-level fluctuators (TLFs) follows the principles reviewed in [21]. The timeline for the experiment is shown schematically in Figure S1. The differential error signal x is measured after each burst (i.e., transfer sequence) of length t_i, repeated consecutively N_i times, for a fixed set of burst lengths $i = 1 \ldots L$.

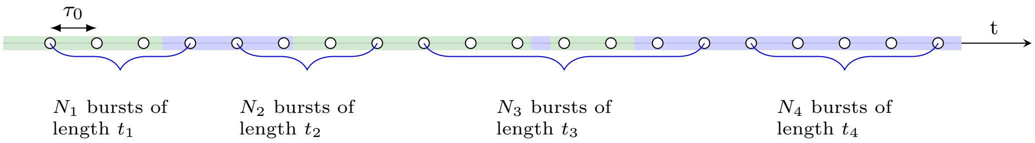

FIG. S1. Illustration of the experimental timeline. Circles denote a single instance of the random walk (one burst). The colors show the state of a single fluctuator (green for 0, blue for 1) in a particular realization of the simulated disorder.

We explore the regime when the duration of one burst, $t_i\, f^{-1}$, is much shorter than the detector-limited time interval τ_0 between the repetitions (e.g., up to a few µs for the former and on the order of a ms for the latter for device A). Switching events in the environment can only contribute significantly on time scales of τ_0 and longer, and are neglected during the short bursts. This means that $P_\pm(\mathrm{t})$ remain fixed during a single instance of the random walk starting at an absolute time t (an integer multiple of τ_0), and the particular value of x is distributed according to Eq. (1) with a particular instance of $P_\pm(\mathrm{t})$. In the statistical models defined in Supplementary Note 9–11, the values of $P_\pm$ are drawn from a Dirichlet distribution after the time τ_0 ("fast fluctuator") or after time $\tau_0\, N_i$ ("slow drift"). Here, in contrast, we generate $P_\pm(\mathrm{t})$ from a continuous-time Markov process characterized by a set of switching rates $\{\Gamma_1, \Gamma_2 \ldots, \Gamma_M\}$ with $\Gamma_m \geq \tau_0^{-1}$.

The value of $P_\pm(\mathrm{t})$ is determined by an average over M two-level fluctuators,

$$P_\pm(\mathrm{t}) = \frac{1}{M} \sum_{m=1}^{M} P_\pm^{(m)} \left[\xi_m(\mathrm{t})\right] , \tag{14}$$

where $\xi_m(\mathrm{t}) = 0$ or 1 is the state of the m-th fluctuator at time t. Each fluctuator is characterized by two modes, $P_\pm^{(m)}[0]$ and $P_\pm^{(m)}[1]$, both drawn once for each full-timeline simulation from a globally fixed Dirichlet distribution. Parameters of the latter are the mean values $\langle P_\pm \rangle$ and the total α_{noise} which determines the level of excess noise. The scatter parameter α_{noise} of the mode distribution is free; it controls the excess noise level.

Switchings $\xi_m \to 1 - \xi_m$ happen randomly with a rate Γ_m (same in both directions) independently of other fluctuators. Hence each fluctuator is described by a 2×2 continuous-time Markov chain transition rate matrix $\left(\begin{smallmatrix} -\Gamma_m & \Gamma_m \\ \Gamma_m & -\Gamma_m \end{smallmatrix}\right)$. An example of a timetrace of one fluctuator switching between two modes is shown by colorboxes in Figure S1.

Two cases are considered:

- $1/f$-noise: $M = 100$, $\Gamma_m = (10^{-8} \ldots 10^{0})\, \tau_0^{-1}$, with $\log \Gamma_m$ chosen randomly from a uniform distribution.

- Single TLF: $M = 1$ and Γ_1 as an adjustable parameter.

In a simulation, N_{tot} values of x are available. For each burst length t_i, the corresponding vector $\mathbf{z}_i$ containing the number of counts for each category of x is compiled in the same way as in experiment. For statistical evaluation of the simulated timetraces, we have binned the simulated counts into five categories, instead of seven as in Eq. (7).

We fixed the set of $L = 42$ burst lengths with $1 \leq t_i \leq 100$ and samples sizes $\{N_1 \ldots N_L\}$ with $N_{\text{tot}} = \sum_{i=1}^{L} N_i = 38\,022\,642$ and $5.00 \times 10^5 \leq N_i \leq 1.40 \times 10^6$ to be the same as for experimental results on device A reported in Figure 2 in the main text.

Similarly, parameters of the Dirichlet distribution for the fluctuator modes are chosen to be comparable to the experimental values, $(\langle P_+ \rangle, \langle P_- \rangle) = (2.10 \times 10^{-5}, 7.00 \times 10^{-5})$.

We quantify the excess noise level by computing directly the relative standard deviation (RSD) of the simulated time traces $P_+(\mathrm{t})$ and $P_-(\mathrm{t})$, and choosing the maximum: $\text{RSD} = \max_{s=+,-} \sigma_s/\mu_s$, where

$$\mu_s := \frac{1}{N_{\text{tot}}} \sum_{n=1}^{N_{\text{tot}}} P_s(n\, \tau_0), \quad \sigma_s^2 := \frac{1}{N_{\text{tot}}} \sum_{n=1}^{N_{\text{tot}}} \left[P_s(n\, \tau_0) - \mu_s\right]^2 . \tag{15}$$

are respectively the mean and the standard deviation of a particular simulated time trace; the time along the data acquisition time-line $\mathrm{t} = n\, \tau_0$ is measured from 0.

SUPPLEMENTARY NOTE 14. NOISE SIMULATION RESULTS

Detection of excess noise

We have run a number of simulations of $1/f$ noise with varying the noise strength parameter α_{noise} from $10^{7.5}$ to $10^{3.6}$ and checked the statistical consistency of the accumulated error counts with the three simple models discussed in the main text: baseline model (no excess noise), "fast fluctuator" and "slow drift".

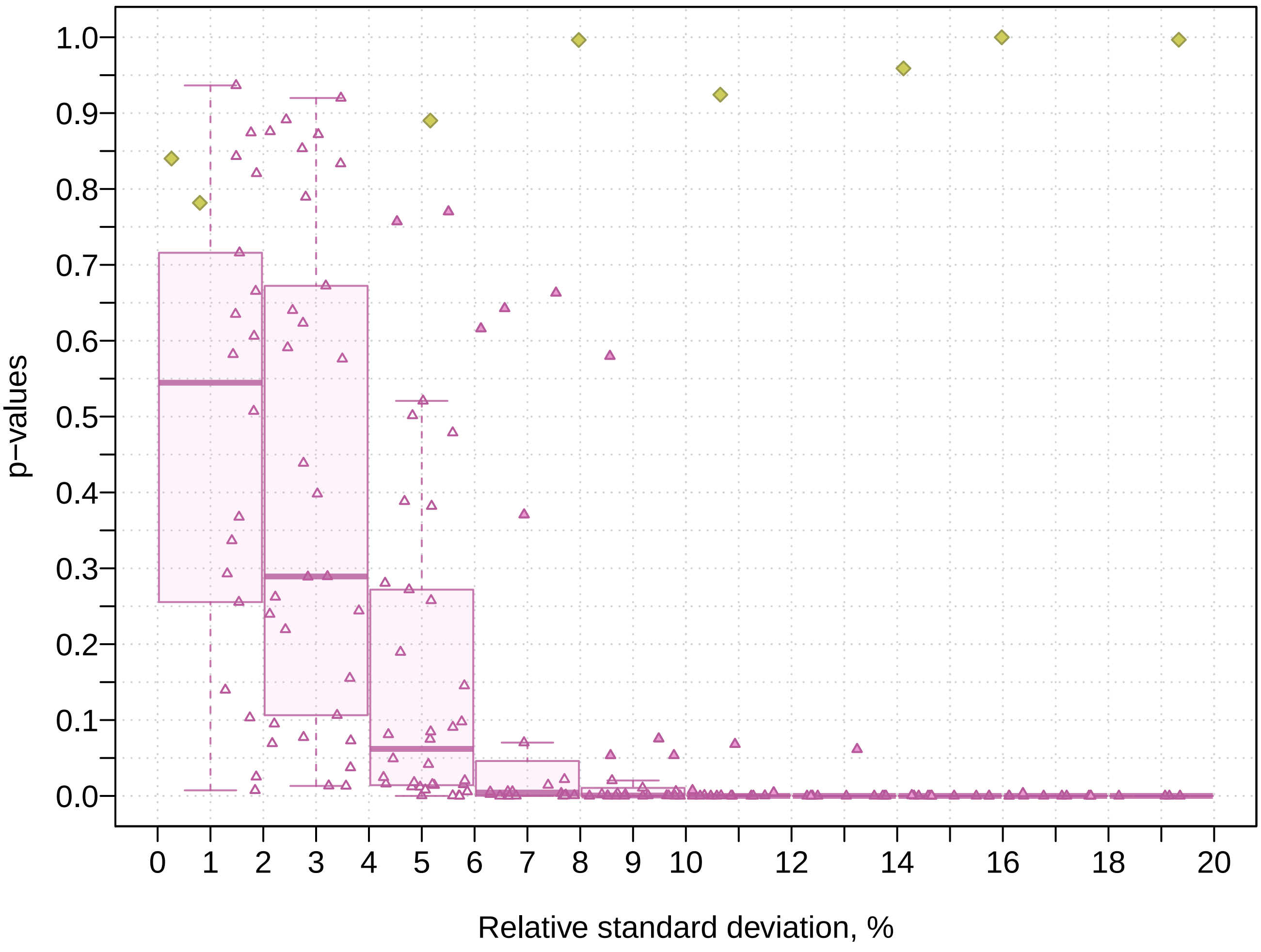

FIG. S2. Results of consistency tests applied to simulated $1/f$ noise for the baseline (triangles) and the "slow drift" (diamonds) excess noise models. Horizontal axis: the relative standard deviation of the simulated timetrace $P_{\pm}(\mathrm{t})$. Triangles: combined p-values returned by the baseline model. Also depicted the box-and-whisker plots of groups of these values, with filled triangles representing outliers in each group. The diamonds depict the combined p-values returned by the "slow drift" test.

The results are plotted in Figure S2. Each symbol represents a simulation; its abscissa equals the RSD of the respective timetrace and its ordinate is the combined p-value obtained via consistency testing of the respective maximum likelihood estimate.

First we consider the baseline model results shown by triangles in Figure S2. For very low noise (essentially constant $P_{\pm}(\mathrm{t})$) the null hypothesis is expected to be true, and the p-values should be scattered approximately uniformly between 0 and 1, while for stronger noise we expect small p to become progressively more likely.

To illustrate this transition more clearly, we have added to Figure S2 box-and-whisker plots of the same dataset, by binning the baseline p-values by the corresponding RSD, i.e., first group with RSD $< 2\%$, second with $2\% \leq$ RSD $< 4\%$ and so on, up to $18\% \leq$ RSD $< 20\%$. The box-and-whisker plots are computed using the standard way, i.e., if Q_1, Q_2, Q_3 are the quartiles of the group and IQR $= Q_3 - Q_1$ is the interquartile range, then the lower and upper limits

of the box are placed at Q_1 and Q_3, respectively, and the horizontal line within the box represents the median Q_2. Furthermore, the lower and upper fences are computed as $Q_1 - 1.5\,\mathrm{IQR}$ and $Q_3 + 1.5\,\mathrm{IQR}$, respectively; if any value in the group is below the lower or above the upper fence, it is considered an outlier and plotted with a filled symbol. Finally, whiskers are drawn so that the upper whisker is located either at the upper fence or the maximal value in the group (whichever is smaller); similarly, the lower whisker is located either at the lower fence or the minimal value in the group (whichever is larger).

As it can be observed in the Figure, the first two groups with RSD up to 4% appear to be consistent with the standard uniform distribution. However, for relative standard deviation above 4% the distribution of the resulting p-values quickly deteriorates and for RSD $\geq$ 6% already the median of the obtained p-values is well below the conventional $p = 0.05$ threshold. We see a clear evidence of a threshold in environmental noise strengths (quantified by RSD in the simulation timetrace): if the noise is too low, the data are consistent with the baseline model. For larger values of RSD, the p-values for the baseline model collapse to very low values, indicating strong rejection of the null hypothesis of no excess noise.

Next we evaluate to what extent the data collected from $1/f$ noise simulations are consistent with single-timescale models discussed in the main text and in Supplementary Note 9 and 11. We have found that for this particular type of noise the behavior of the "fast fluctuator"' test offers only a marginal improvement over the baseline model, hence the corresponding p-values are not plotted.

To the contrary, the "slow drift" model is generally consistent with the simulated data even if the excess noise level is high, provided that parameters of the Dirichlet distribution in the "slow-drift model" (see Eq. 12) are chosen appropriately.

In Figure S2 the diamonds show the combined p-values for simulated noise data tested against the "slow drift" model using the Monte Carlo estimation procedure of Supplementary Note 12. Here we have used $\max_{s=+,-} \sigma_s$ computed from the simulated timetrace $P_\pm(\mathrm{t})$ to set *a priori* the concentration parameter $\alpha_\bullet$ of the Dirichlet distribution used in the "slow drift" test. Despite the value of $\alpha_\bullet$ not being optimized (and hence, potentially biasing the test), the results indicate that the "slow drift" model for the data is not rejected: the p-values are high for the whole range of noise levels considered.

Example of statistical methodology applied to the simulated data

Here we illustrate in more detail application of statistical tests to a particular simulated timetrace of $1/f$ noise model. The simulation has used $\alpha_{\mathrm{noise}} = 10^{5.2}$ and $\langle P_+ \rangle = 2.12 \times 10^{-5}$, $\langle P_- \rangle = 6.90 \times 10^{-5}$.

The estimated mean μ_s and the standard deviation σ_s of the simulated $P_\pm(\mathrm{t})$ values are $\mu_+ = 2.11 \times 10^{-5}, \mu_- = 6.94 \times 10^{-5}$ and $\sigma_+ = 7.72 \times 10^{-7}, \sigma_- = 1.37 \times 10^{-6}$, which gives the relative standard deviation RSD = 3.66%. The simulated $P_\pm(\mathrm{t})$ values are well approximated by the normal distribution with the respective parameters; see the histograms of $P_\pm(\mathrm{t})$ in Figure S3b.

Next we describe how the spectrum of the simulated noise was analyzed. Let $R = 2^{18}$, $K = \lfloor \frac{N_{\mathrm{tot}}}{R} \rfloor = 145$. Given the signal $P_s(n\,\tau_0)$, $n = 1, 2, \ldots, N_{\mathrm{tot}}$, where $s \in \{-, +\}$, its power spectral density (PSD) is estimated by splitting the signal into K non-overlapping segments of length R (the signal components with $n > KR$ are discarded) and computing the periodogram for each segment and averaging the results for the K segments. Each periodogram is found by computing the squared magnitude of the discrete Fourier transform and dividing the result by R; moreover, the values at all frequencies except 0 and 0.5 are doubled since the one-sided periodogram is considered.

In Figure S3a we show the PSD estimates of the simulated timetrace which show the signature $1/f$ roll-off.

Next, we analyze the simulated sample in the same way as the experimental data for device A, c.f. Figure 2 in the main text.

For the baseline model, the maximum likelihood gives the estimate $(P_+, P_-) = \left(2.07 \times 10^{-5}, 6.96 \times 10^{-5}\right)$, which correlates well with the underlying $\mu_\pm \pm \sigma_\pm$. Next, we employ Fisherian significance tests separately for each block of N_i counts for a fixed burst length t_i to define consistency regions of p-value greater than 0.05 in the parameter space (P_+, P_-) where the baseline model cannot be rejected at this significance level. These quasielliptic consistency regions are depicted in Figure S3b; again, their overlap is only partial. Fisher's meta-analysis method yields the combined p-value 0.04, thus the baseline model is nominally rejected.

The simulated data then were tested against the "slow drift" model with the optimized (as explained in Supplementary Note 12) parameters $\boldsymbol{\alpha}^* = (5.01 \times 10^3, 7.20 \times 10^7, 1.49 \times 10^3)$. The cumulative distribution of the obtained p-values for each sequence length is shown in Figure S3c; it minimizes the empirical distance measure (13) and is close to being uniform. Quantitatively, the combined p-value equals 0.45, implying that the simulated data are not

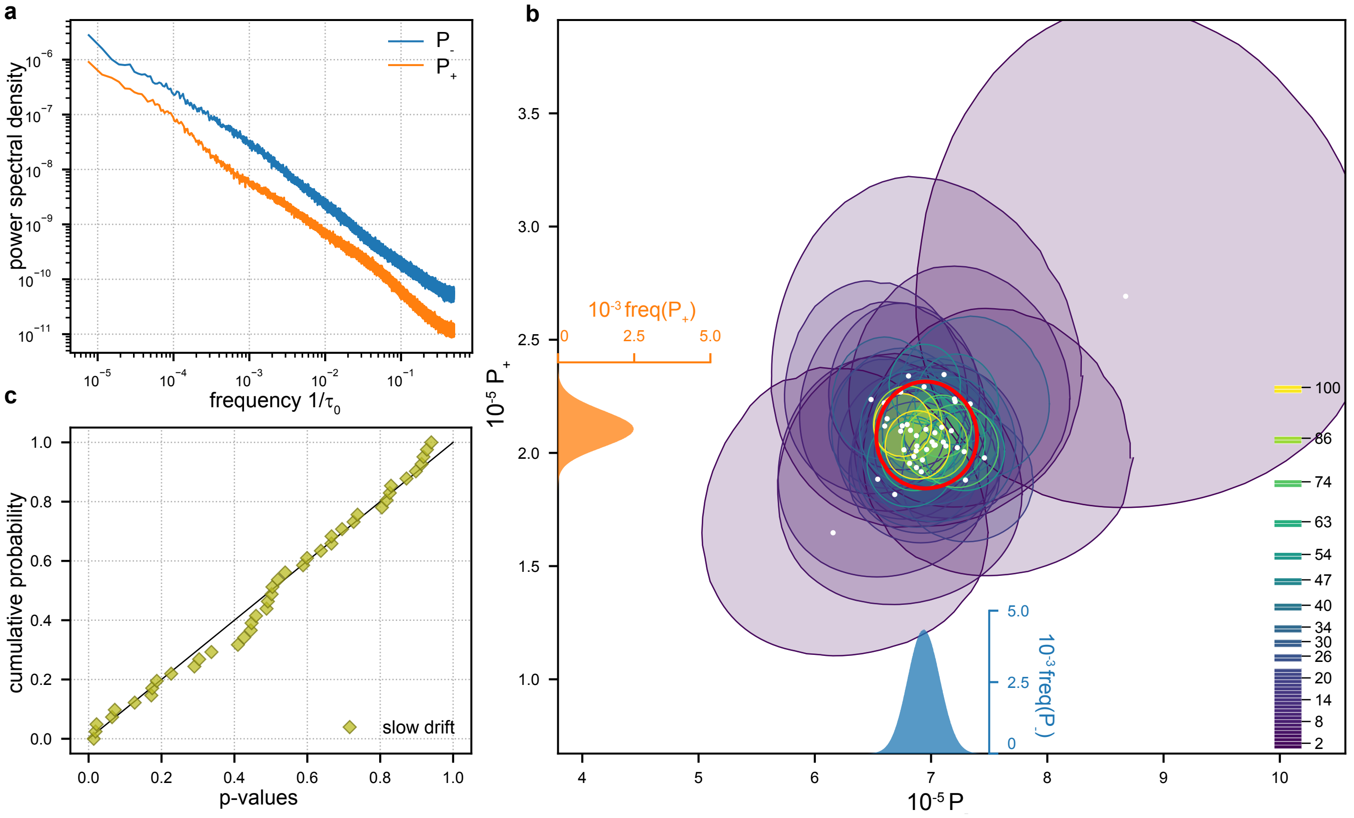

FIG. S3. (a) The PSD estimates of the simulated timetrace. (b) White dots: maximum-likelihood estimates of $P_\pm$ in the baseline model for each sequence length separately. Also $p > 0.05$ consistency regions are shown (color indicating the sequence length). The histograms of the simulated $P_\pm(\mathrm{t})$ values are depicted on the axes of the plot. (c) Empirical cumulative distribution of p-values for the slow-drift model in comparison to the uniform distribution (black line).

inconsistent with the slow-drift model.

The standard deviations of $P_\pm$ in the Dirichlet distribution Dir($\boldsymbol{\alpha}^*$) are 5.36×10^{-7} and 9.83×10^{-7}, respectively, producing the 1σ estimates

$$P_+ = (2.07 \pm 0.05) \times 10^{-5}, \quad P_- = (6.96 \pm 0.10) \times 10^{-5} \quad (\text{“slow drift” estimate})$$

which compare well with $\mu_\pm \pm \sigma_\pm$ accessible for the simulated noise,

$$P_+ = (2.11 \pm 0.08) \times 10^{-5}, \quad P_- = (6.94 \pm 0.14) \times 10^{-5} \quad (\text{simulated noise})$$

Simulations of a single fluctuator

We have explored the excess noise model with a single fluctuator ($M = 1$) with the switching rate Γ_1 varied in simulations from $10^{-6}\tau_0^{-1}$ to τ_0^{-1}. The distribution of $P_\pm(\mathrm{t})$ in this case is bimodal, with the two modes, $P_\pm^{(1)}[0]$ and $P_\pm^{(1)}[1]$, chosen randomly before the start of the simulation. The corresponding noise power spectral density is a Lorentzian that crosses over from a constant to $1/f^2$ at frequency $f \sim \Gamma_1$.

With respect to the baseline and the “slow drift” statistical tests, the single-fluctuator model behaves similarly to the $1/f$ case considered above, regardless of the choice of Γ_1: a sufficiently large distance between the two modes generates counts inconsistent with the baseline, but compatible with the “slow drift” model with an appropriately chosen $\Delta P_\pm$.

The results of “fast flcutuator” tests do, in general, depend on the switching speed Γ_1: for fast $\Gamma_1 \sim \tau_0^{-1}$ the simulated data typically are consistent with the “fast fluctuator” model, while for slower $\Gamma_1 \ll \tau_0^{-1}$ the simulated data reject the “fast fluctuator” noise model.

These observations can be understood considering the characteristic frequencies to which our statistical tests are sensitive: "slow drift" probes the low-frequency part of the noise spectrum (switching rates on the order of $1/(N_i\tau_0) \sim 10^{-5}\tau_0^{-1}$) while the "fast fluctuator" tests relatively high frequencies ($\sim \tau_0^{-1}$), at which the noise from a slow ($\Gamma_1 \ll \tau^{-1}$) single fluctuator is sufficiently suppressed by the $1/f^2$ roll-off.

SUPPLEMENTARY NOTE 15. SLOW-DRIFT MODEL: VARIANCE OF RANDOM WALKER'S POSITION

In this section we derive the formula for the variance of the random walker's position Δx^2 used in the concluding part of the main text.

Consider repeatedly sampling random walker's position in the slow-drift model; we are interested in the sample variance. However, the samples are correlated, since the model assumes using the same parameter $\boldsymbol{\theta} \sim \mathrm{Dir}(\boldsymbol{\alpha})$ for several (N) successive random walks. Henceforth, we consider the following scenario: draw $\boldsymbol{\theta}^{(1)} \sim \mathrm{Dir}(\boldsymbol{\alpha})$ and run t steps of the random walk with step probabilities $\boldsymbol{\theta}^{(1)}$ for N times; let $X_1^{(1)}$, $X_2^{(1)}$, …, $X_N^{(1)}$ be the random walker's position after the respective random walk has been completed. Afterwards, the step probabilities reset, i.e., a new parameter $\boldsymbol{\theta}^{(2)} \sim \mathrm{Dir}(\boldsymbol{\alpha})$ is independently drawn, and N times random walk of t steps is run with step probabilities $\boldsymbol{\theta}$, resulting in random variables $X_1^{(2)}$, $X_2^{(2)}$, …, $X_N^{(2)}$. The process is continued until, say, K blocks $\mathbf{X}^{(k)} := \left(X_1^{(k)}, X_2^{(k)}, \ldots, X_N^{(k)}\right)$, $k = 1, 2, \ldots, K$, are obtained. It is important to stress that

1. the blocks $\mathbf{X}^{(1)}, \mathbf{X}^{(2)}, \ldots, \mathbf{X}^{(K)}$ are assumed to be pairwise independent;
2. within each block, variables $X_i^{(k)}$ and $X_j^{(k)}$, $i \neq j$, $k = 1, 2, \ldots, K$, are assumed to be conditionally independent given $\boldsymbol{\theta}^{(k)}$, i.e.,

$$(X_i^{(k)} \perp\!\!\!\perp X_j^{(k)}) \mid \boldsymbol{\theta}^{(k)}.$$

Let $M := KN$; we are interested in the quantity

$$S := \frac{1}{M} \sum_{k=1}^{K} \sum_{i=1}^{N} \left(X_i^{(k)} - \overline{X}\right)^2,$$

where $\overline{X} := \frac{1}{M} \sum_{k=1}^{K} \sum_{i=1}^{N} X_i^{(k)}$. In particular, the task is to find $\Delta x^2 := \mathrm{E}(S)$ in the $K \to \infty$ limit.

Claim 3. *The expectation $\Delta x^2 = \mathrm{E}(S)$ in the $P_\pm \ll 1$, $K \to \infty$ limit satisfies*

$$\Delta x^2 = \frac{A}{1 + \alpha_\bullet} \left(\frac{t\alpha_\bullet(M-1)}{M} + \frac{t^2(K-1)}{K}\right) \approx \left(\langle P_+\rangle + \langle P_\pm\rangle\right) t + \left(\Delta P_+^2 + \Delta P_-^2\right) t^2,$$

where

$$\begin{aligned}
&A := (\tilde{\alpha}_{-1} + \tilde{\alpha}_1) - (\tilde{\alpha}_{-1} - \tilde{\alpha}_1)^2\,; \\
&\tilde{\alpha}_{\pm 1} := \frac{\alpha_{\pm 1}}{\alpha_\bullet}; \quad \alpha_\bullet := \alpha_{-1} + \alpha_0 + \alpha_1; \\
&\langle P_\pm \rangle := \mathrm{E}(P_\pm) = \tilde{\alpha}_{\pm 1}; \\
&\Delta P_\pm^2 := \mathrm{Var}(P_\pm) = \frac{\tilde{\alpha}_{\pm 1}(1 - \tilde{\alpha}_{\pm 1})}{1 + \alpha_\bullet}.
\end{aligned}$$

Proof. We start by noting that each block $\mathbf{X}^{(k)}$ is an independent observation of the random variable $\mathbf{X} = (X_1, X_2, \ldots, X_N)$, where $\mathbf{X}$ is obtained by the process above with $K = 1$. Let us define a random variable $Y = \frac{1}{N}(X_1 + \ldots + X_N)$, then there are K iid variables $Y_1, \ldots, Y_K \sim Y$ corresponding to the blocks $\mathbf{X}^{(1)}$, …, $\mathbf{X}^{(K)}$. We can express

$$S = \frac{1}{M} \sum_{i,k} \left(X_i^{(k)}\right)^2 - \frac{2}{M} \sum_{i,k} \left(X_i^{(k)}\right) \overline{X} + \overline{X}^2 = \frac{1}{M} \sum_{i,k} \left(X_i^{(k)}\right)^2 - \overline{X}^2$$

and

$$\overline{X} = \frac{1}{K}(Y_1 + Y_2 + \ldots + Y_K).$$

Since $X_i^{(k)}$ are identically distributed, we have

$$\mathrm{E}\left(\frac{1}{M}\sum_{k=1}^{K}\sum_{i=1}^{N}\left(X_i^{(k)}\right)^2\right) = \frac{M}{M}\cdot \mathrm{E}(X_1^2) = \mathrm{Var}(X_1) + \mathrm{E}(X_1)^2;$$

since $Y_1, \ldots, Y_K$ are iid, we have

$$(Y_1 + Y_2 + \ldots + Y_K)^2 = \sum_{k=1}^{K} Y_k^2 + \sum_{k=1}^{K}\sum_{\substack{l=1\\ l\neq k}}^{K} Y_k Y_l$$

and

$$\mathrm{E}\left(\overline{X}^2\right) = \frac{1}{K}\,\mathrm{E}(Y^2) + \frac{K-1}{K}\,\mathrm{E}(Y)^2 = \frac{1}{K}\,\mathrm{Var}(Y) + \mathrm{E}(Y)^2.$$

Consequently,

$$\Delta x^2 = \mathrm{Var}(X_1) + \mathrm{E}(X_1)^2 - \frac{1}{K}\,\mathrm{Var}(Y) - \mathrm{E}(Y)^2.$$

Let us show that

$$\mathrm{E}(X_1) = t\,(\tilde{\alpha}_1 - \tilde{\alpha}_{-1}) \tag{16}$$

$$\mathrm{Var}(X_1) = \frac{At\alpha_\bullet}{1+\alpha_\bullet} + \frac{At^2}{1+\alpha_\bullet}, \tag{17}$$

$$\mathrm{E}(Y) = \mathrm{E}(X_1), \tag{18}$$

$$\mathrm{Var}(Y) = \frac{At\alpha_\bullet}{N(1+\alpha_\bullet)} + \frac{At^2}{1+\alpha_\bullet}, \tag{19}$$

then we will arrive at

$$\Delta x^2 = \mathrm{Var}(X_1) - \frac{1}{K}\,\mathrm{Var}(Y) = \frac{At\alpha_\bullet(M-1)}{M(1+\alpha_\bullet)} + \frac{At^2(K-1)}{K(1+\alpha_\bullet)},$$

as desired.

Equalities (16) **and** (17)**.** If $\boldsymbol{\theta} = (P_-, P_0, P_+)$ is a fixed parameter, then the position of the random walker after t steps with step probabilities given by $\boldsymbol{\theta}$ is given by $\delta_1 + \ldots + \delta_t$, where $\delta_i \sim \delta$ are iid and

$$\delta = \begin{cases} -1, & \text{with prob. } P_-, \\ 0, & \text{with prob. } P_0, \\ +1, & \text{with prob. } P_+. \end{cases}$$

Consequently, for the Dirichlet-distributed $\boldsymbol{\theta}$ the conditional expectation / variance $\mathrm{E}(X_1 \mid \boldsymbol{\theta})$ and $\mathrm{Var}(X_1 \mid \boldsymbol{\theta})$ satisfy

$$\begin{aligned} \mathrm{E}(X_1 \mid \boldsymbol{\theta}) &= t\,(P_+ - P_-) \\ \mathrm{Var}(X_1 \mid \boldsymbol{\theta}) &= t\,(P_+ + P_-) - t\,(P_+ - P_-)^2\,. \end{aligned}$$

By the laws of total expectation / variance, we obtain (16) and (17):

$$\begin{aligned} \mathrm{E}(X_1) &= \mathrm{E}\left(\mathrm{E}(X_1 \mid \boldsymbol{\theta})\right) = t(\tilde{\alpha}_1 - \tilde{\alpha}_{-1}) \\ \mathrm{Var}(X_1) &= \mathrm{E}(\mathrm{Var}(X_1 \mid \boldsymbol{\theta})) + \mathrm{Var}(\mathrm{E}(X_1 \mid \boldsymbol{\theta})) \\ &= t\,\mathrm{E}\left(P_+ + P_- + 2P_+P_- - P_+^2 - P_-^2\right) + t^2\,\mathrm{Var}\left(P_+ - P_-\right) \\ &= \frac{t\alpha_\bullet A}{1+\alpha_\bullet} + \frac{t^2 A}{1+\alpha_\bullet}. \end{aligned}$$

To show the last equality, recall the relevant properties of $(P_-, P_0, P_+) \sim \mathrm{Dir}(\boldsymbol{\alpha})$:

$$\begin{aligned} &\mathrm{E}(P_\pm) = \tilde{\alpha}_{\pm 1};\\ &\mathrm{Var}(P_\pm) = \frac{\tilde{\alpha}_{\pm 1}(1-\tilde{\alpha}_{\pm 1})}{1+\alpha_\bullet};\\ &\mathrm{Cov}(P_+; P_-) = \frac{-\tilde{\alpha}_{-1}\tilde{\alpha}_1}{1+\alpha_\bullet}. \end{aligned}$$

Thus

$$\mathrm{Var}\left(P_+ - P_-\right) = \mathrm{Var}\left(P_+\right) + \mathrm{Var}\left(P_-\right) - 2\,\mathrm{Cov}(P_+; P_-) = \frac{A}{1+\alpha_\bullet}$$

and

$$\begin{aligned} &\mathrm{E}\left(P_+ + P_- + 2P_+P_- - P_+^2 - P_-^2\right)\\ &= \mathrm{E}\left(P_+ + P_-\right) + 2\left(\mathrm{Cov}(P_+; P_-) + \mathrm{E}(P_+)\,\mathrm{E}(P_-)\right) - \left(\mathrm{Var}\left(P_+\right) + \mathrm{E}(P_+)^2\right) - \left(\mathrm{Var}\left(P_-\right) + \mathrm{E}(P_-)^2\right)\\ &= \mathrm{E}\left(P_+ + P_-\right) - \left(\mathrm{E}\left(P_+ - P_-\right)\right)^2 - \mathrm{Var}\left(P_+ - P_-\right)\\ &= A - \frac{A}{1+\alpha_\bullet} = \frac{\alpha_\bullet A}{1+\alpha_\bullet}. \end{aligned}$$

Equality (18)**.** This equality trivially follows from the definition of Y and the fact that X_i are identically distributed:

$$\mathrm{E}(Y) = \frac{1}{N}\,\mathrm{E}(X_1 + \ldots + X_N) = \frac{N}{N}\,\mathrm{E}(X_1).$$

Equality (19)**.** The variance of the sum $X_1 + \ldots + X_N$ is the sum of the covariances:

$$\mathrm{Var}(X_1 + \ldots + X_N) = \sum_{i=1}^{N} \mathrm{Var}(X_i) + 2 \sum_{1 \le i < j < N} \mathrm{Cov}(X_i, X_j)$$

However, X_i are identically distributed, therefore

$$\mathrm{Var}(Y) = \frac{1}{N^2}\left(N\,\mathrm{Var}(X_1) + (N^2 - N)\,\mathrm{Cov}(X_1, X_2)\right) \tag{20}$$

$\mathrm{Var}(X_1)$ is given by (17), it remains to find $\mathrm{Cov}(X_1, X_2)$. By the law of total covariance,

$$\mathrm{Cov}(X_1, X_2) = \mathrm{E}\left(\mathrm{Cov}\left(X_1, X_2 \mid \boldsymbol{\theta}\right)\right) + \mathrm{Cov}\left(\mathrm{E}\left(X_1 \mid \boldsymbol{\theta}\right), \mathrm{E}\left(X_2 \mid \boldsymbol{\theta}\right)\right)$$

Since X_1, X_2 are conditionally independent given $\boldsymbol{\theta}$, the conditional covariance vanishes: $\mathrm{Cov}\left(X_1, X_2 \mid \boldsymbol{\theta}\right) = 0$. Moreover, as X_1, X_2 are identically distributed,

$$\mathrm{E}\left(X_1 \mid \boldsymbol{\theta}\right) = \mathrm{E}\left(X_2 \mid \boldsymbol{\theta}\right) = t\left(P_+ - P_-\right),$$

thus

$$\mathrm{Cov}(X_1, X_2) = t^2\,\mathrm{Var}\left(P_+ - P_-\right) = \frac{t^2 A}{1+\alpha_\bullet}.$$

We arrive at

$$\mathrm{Var}(Y) = \frac{1}{N}\left(A \cdot \frac{t\alpha_\bullet + t^2}{1+\alpha_\bullet} + (N-1)\frac{t^2 A}{1+\alpha_\bullet}\right) = \frac{At\alpha_\bullet}{N(1+\alpha_\bullet)} + \frac{At^2}{1+\alpha_\bullet},$$

which concludes the proof. □

SUPPLEMENTARY NOTE 16. PROOF FOR SPREAD CONDITION

This section contains the proof for the spread condition shown in (2) in the main text (restated here for convenience).

Claim 4. *For any random process on the line with steps of length 1, the spread condition*

$$\sum_{y=-\infty}^{x-1} p_y^t \leq \sum_{y=-\infty}^{x} p_y^{t+1} \leq \sum_{y=-\infty}^{x+1} p_y^t \quad \text{for all } x, \tag{21}$$

must be satisfied.

Proof. If the random process is at location $y \leq x-1$ after t time steps, it must be at a location $y' \leq x$ after $t+1$ time steps. Hence, $\sum_{y=-\infty}^{x-1} p_y^t \leq \sum_{y=-\infty}^{x} p_y^{t+1}$.

On the other hand, if the process is at a location $y \leq x$ after $t+1$ time steps, it has been at a location $y' \leq x+1$ after t time steps. Hence, $\sum_{y=-\infty}^{x} p_y^{t+1} \leq \sum_{y=-\infty}^{x+1} p_y^t$. □

We note that the claim applies not just to Markov processes but to any random process that can move at most distance 1 in one time step. For example, it applies to processes where the transition probabilities depend not just on the current location but also on locations in previous time steps.

Claim 5. *If two probability distributions $(p_x^t)_{x\in\mathbb{Z}}$ and $(p_x^{t+1})_{x\in\mathbb{Z}}$ satisfy the spread condition* (21)*, then there exists a set of transition probabilities $P_{\pm 1}^{(x,t)}$ such that $(p_x^{t+1})_{x\in\mathbb{Z}}$ is generated from $(p_x^t)_{x\in\mathbb{Z}}$ by a Markov process*

$$p_x^{t+1} = P_{+1}^{(x-1,t)} p_{x-1}^t + \left(1 - P_{+1}^{(x,t)} - P_{-1}^{(x,t)}\right) p_x^t + P_{-1}^{(x+1,t)} p_{x+1}^t, \quad x \in \mathbb{Z}.$$

Proof. Let $q_x^t = \sum_{y=-\infty}^{x} p_y^t$; then the spread condition is equivalent to

$$q_{x-1}^t \leq q_x^{t+1} \leq q_{x+1}^t, \quad \text{for all } x \in \mathbb{Z}.$$

Consider a Markov process in which the probability of moving left from a location x at time t is defined by

$$P_{-1}^{(x,t)} = \begin{cases} 0 & \text{if } q_{x-1}^t \geq q_{x-1}^{t+1} \\ \frac{1}{p_x^t}\left(q_{x-1}^{t+1} - q_{x-1}^t\right) & \text{otherwise,} \end{cases}$$

and the probability of moving right is defined by

$$P_{+1}^{(x,t)} = \begin{cases} 0 & \text{if } q_x^t \leq q_x^{t+1} \\ \frac{1}{p_x^t}\left(q_x^t - q_x^{t+1}\right) & \text{otherwise.} \end{cases}$$

The probability to stay at x is defined as $P_0^{(x,t)} := 1 - P_{+1}^{(x,t)} - P_{-1}^{(x,t)}$. Notice that the spread condition implies

$$q_{x-1}^{t+1} - q_{x-1}^t \leq q_x^t - q_{x-1}^t = p_x^t \quad \text{and} \quad q_x^t - q_x^{t+1} = q_{x-1}^t + p_x^t - q_x^{t+1} \leq p_x^t,$$

thus $P_{-1}^{(x,t)} \leq 1$ and $P_{+1}^{(x,t)} \leq 1$. Finally, we have $P_0^{(x,t)} \geq 0$. To see that, it suffices to consider the case when $P_{+1}^{(x,t)}$ and $P_{-1}^{(x,t)}$ are both positive; then we have to show

$$\left(q_x^t - q_x^{t+1}\right) + \left(q_{x-1}^{t+1} - q_{x-1}^t\right) \leq p_x^t.$$

However, this inequality clearly holds, since

$$\left(q_x^t - q_x^{t+1}\right) + \left(q_{x-1}^{t+1} - q_{x-1}^t\right) = \left(q_x^t - q_{x-1}^t\right) + \left(q_{x-1}^{t+1} - q_x^{t+1}\right) = p_x^t - p_x^{t+1} \leq p_x^t.$$

Therefore we have defined valid transition probabilities.

To see that this process produces $(p_x^{t+1})_{x\in\mathbb{Z}}$, let $\overline{q}_x^{t+1}$ be the probability of being at a location $x' \leq x$ after applying these transition probabilities to the distribution $(p_x^t)_{x\in\mathbb{Z}}$. We show that $\overline{q}_x^{t+1} = q_x^{t+1}$ for all x, thus the probability of being at any particular x_0 equals $q_{x_0}^{t+1} - q_{x_0-1}^{t+1} = p_{x_0}^{t+1}$. We consider two cases.

1. If $q_x^t \leq q_x^{t+1}$, we have $P_{+1}^{(x,t)} = 0$ and

$$\overline{q}_x^{t+1} = q_x^t + p_{x+1}^t P_{-1}^{(x+1,t)} = q_x^t + \left(q_x^{t+1} - q_x^t\right) = q_x^{t+1}.$$

2. If $q_x^t > q_x^{t+1}$, we have $P_{-1}^{(x+1,t)} = 0$ and

$$\overline{q}_x^{t+1} = q_{x-1}^t + p_x^t \left(1 - P_{+1}^{(x,t)}\right) = q_{x-1}^t + p_x^t - \left(q_x^t - q_x^{t+1}\right) = q_x^{t+1},$$

which concludes the proof. □

A consequence of these two claims is that, given just the probabilities p_x^t, we cannot distinguish whether they come from a (possibly non-stationary, not translation-invariant) Markov process or from a more general process that moves at most distance 1 in one time step. (In the second case, the spread condition will be satisfied and then, because of Claim 5, there will be a time and location dependent Markov process that gives the same p_x^t.)

REFERENCES

* To whom correspondence should be addressed: niels.ubbelohde@ptb.de

[1] Zhang, X.-K., Wan, J., Lu, J.-J. & Xu, X.-P. Recurrence and Pólya Number of General One-Dimensional Random Walks. *Commun. Theor. Phys.* **56**, 293–296 (2011).

[2] Shimizu, K. & Yanagimoto, T. The inverse trinomial distribution. *Jpn. J. Appl. Phys.* **20**, 89–96 (1991). In Japanese.

[3] Aoyama, K. & Shimizu, K. A generalization of the inverse trinomial. Tech. Rep., Keio University. Department of Mathematics (2005). KSTS/RR-05/003.

[4] NIST Digital Library of Mathematical Functions, Release 1.0.27 of 2020-06-15. URL `http://dlmf.nist.gov/`. F. W. J. Olver, A. B. Olde Daalhuis, D. W. Lozier, B. I. Schneider, R. F. Boisvert, C. W. Clark, B. R. Miller, B. V. Saunders, H. S. Cohl, and M. A. McClain, eds.

[5] Erdélyi, A., Magnus, W., Oberhettinger, F. & Tricomi, F. G. *Higher Transcendental Functions. Vol. I* (McGraw-Hill Book Company, Inc., New York-Toronto-London, 1953).

[6] Eichstädt, S. & Wilkens, V. GUM2DFT—a software tool for uncertainty evaluation of transient signals in the frequency domain. *Meas. Sci. Technol.* **27**, 055001 (2016).

[7] Christensen, R. Testing Fisher Neyman Pearson and Bayes. *Am. Stat.* **59**, 121–126 (2005).

[8] Conover, W. J. A Kolmogorov Goodness-of-Fit Test for Discontinuous Distributions. *J. Am. Stat. Assoc.* **67**, 591–596 (1972).

[9] Smith, P. J., Rae, D. S., Manderscheid, R. W. & Silbergeld, S. Approximating the Moments and Distribution of the Likelihood Ratio Statistic for Multinomial Goodness of Fit. *J. Am. Stat. Assoc.* **76**, 737–740 (1981).

[10] Cressie, N. & Read, T. R. C. Multinomial Goodness-of-Fit Tests. *J. R. Stat. Soc. Ser. B Methodol.* **46**, 440–464 (1984).

[11] Jann, B. Multinomial goodness–of–fit: Large–sample tests with survey design correction and exact tests for small samples. *SJ* **08**, 122584 (2008).

[12] Barnard, G. A. Discussion of The spectral analysis of point processes by M. S. Bartlett. *J. R. Stat. Soc. Ser. B Methodol.* **25**, 294 (1963).

[13] Hope, A. C. A. A Simplified Monte Carlo Significance Test Procedure. *J. R. Stat. Soc. Ser. B Methodol.* **30**, 582–598 (1968).

[14] Besag, J. Simple Monte Carlo P-Values. In Page, C. & LePage, R. (eds.) *Computing Science and Statistics*, 158–162 (Springer New York, New York, NY, 1992).

[15] Borenstein, M., Hedges, L. V., Higgins, J. P. T. & Rothstein, H. R. *Meta-Analysis Methods Based on Direction and p-Values*, chap. 36, 325–330 (John Wiley & Sons, Chichester, 2009).

[16] Fisher, R. A. *Statistical methods for research workers* (Oliver & Boyd, Edinburgh, 1932), fourth ed. - revised and enlarged edn.

[17] Winkler, A. M. *et al.* Non-parametric combination and related permutation tests for neuroimaging. *Hum. Brain Mapp.* **37**, 1486–1511 (2016).

[18] Mielke Jr, P. W., Johnston, J. E. & Berry, K. J. Combining Probability Values from Independent Permutation Tests: A Discrete Analog of Fishers Classical Method. *Psychol. Rep.* **95**, 449–458 (2004).

[19] Ng, K. W., Tian, G.-L. & Tang, M.-L. *Dirichlet and related distributions: Theory methods and applications.* (John Wiley & Sons, Hoboken, 2011).

[20] Johnson, N. L., Kotz, S. & Balakrishnan, N. *Discrete multivariate distributions.* Wiley series in probability and statistics (Wiley, New York, 1997).

[21] Paladino, E., Galperin, Y. M., Falci, G. & Altshuler, B. L. $1/f$ noise: Implications for solid-state quantum information. *Rev. Mod. Phys.* **86**, 361–418 (2014).
